# Digital Image Correlation and Numerical Analysis of Mechanical Behavior in Photopolymer Resin Lattice Structures

**DOI:** 10.3390/ma18020384

**Published:** 2025-01-16

**Authors:** Arkadiusz Popławski, Paweł Bogusz, Maciej Grudnik

**Affiliations:** Faculty of Mechanical Engineering, Military University of Technology, Kaliskiego 2 St., 00-908 Warsaw, Poland; arkadiusz.poplawski@wat.edu.pl (A.P.); maciej.grudnik@student.wat.edu.pl (M.G.)

**Keywords:** lattice structures, 3D printing, experimental research, Digital Image Correlation method, SAMP-1 model

## Abstract

Cellular structures are increasingly utilized in modern engineering due to their exceptional mechanical and physical properties. In this study, the deformation and failure mechanisms of two energy-efficient lattice structures—hexagonal honeycomb and re-entrant honeycomb—were investigated. These structures were manufactured using additive stereolithography with light-curable Durable Resin V2. The experimental testing of the topologies under two perpendicular loading directions employed the 3D Digital Image Correlation (DIC) system to capture strain fields and deformation patterns, providing insights into structural behavior and failure mechanisms. The unit cells of the topologies were scaled up to enable precise optical measurements while preserving their structural interaction characteristics. Numerical simulations, conducted using the SAMP-1 material model in LS-DYNA and calibrated with tensile and compression test data, accurately replicated the behavior of the studied topologies and demonstrated good agreement with experimental results. The hexagonal structure, loaded along axis 2, showed the best fit, with deviations within 5%, while the re-entrant honeycomb structure exhibited weaker yet reasonable agreement. By integrating experimental and numerical approaches, the research validates the SAMP-1 model’s predictive capabilities for lattice structures and provides a framework for analyzing energy-absorbing lattice topologies.

## 1. Introduction

Cellular structures are gaining significant popularity in modern engineering industries due to their advantageous mechanical and physical properties. In recent years, their development has accelerated worldwide. This extensive and diverse group includes foamed materials, particularly metallic foams. However, their structure is generally stochastic, meaning that the pores are randomly distributed throughout the material [1,2].

The advancement of additive 3D printing methods has enabled the development and creation of so-called lattice structures. These structures are typically formed by repeating a unit cell, which serves as the basic building block for the entire structure. Unlike metallic foams, lattice structures are highly specialized materials characterized by diverse and intricate patterns, which may be regular, irregular, or stochastic [3,4,5,6]. By deliberately positioning material and designing structural layouts, these systems allow for the creation of large-scale frameworks with tailored mechanical properties. Common designs include rod-based topologies that intersect in multiple directions for effective load distribution. More advanced geometries, such as gyroid surfaces with seamless curvature, diamond-like patterns for optimized strength-to-weight ratios, graded structures for variable mechanical properties, and triply periodic minimal surfaces (TPMS) with repeating complex forms, further expand their applicability [7,8,9,10].

Lattice structures are particularly well-suited for 3D printing through additive manufacturing techniques. Their ability to minimize the mass of individual components often translates into shorter printing times and lower material consumption, making them highly efficient in most scenarios [11,12,13].

Although additive 3D printing methods offer numerous advantages for lattice structure development, they also have limitations that affect quality and accuracy. These include satellite droplets leading to material residues, nozzle clogging causing inconsistent material flow, material heterogeneity, poor adhesion between layers reducing mechanical strength, and shrinkage during cooling of certain materials resulting in potential deformation. Such limitations impact the mechanical properties and consistency of printed components.

The ongoing evolution of additive manufacturing, particularly 3D printing, has significantly advanced despite inherent limitations. Lattice structures play a pivotal role in engineering and material sciences, combining lightweight characteristics, flexibility, and remarkable strength [14,15]. Their repeating and interwoven designs ensure that stresses are distributed uniformly, enhancing their effectiveness in supporting loads while conserving material usage [16,17]. These characteristics are particularly critical in fields such as aviation, automotive design, and construction, where reducing weight is crucial without compromising on durability. Furthermore, these structures are designed to efficiently manage energy absorption and dissipation, which makes them highly effective in mitigating shocks and impacts [18,19,20]. Detailed analysis of their mechanical performance is essential to optimize their implementation in varied technical contexts [21,22].

Lattice configurations are typically classified based on their internal arrangements, such as tensile-dominated or bending-dominated systems. Maxwell’s stability criterion often serves as a tool for identifying these categories. Bending-dominated configurations excel at absorbing energy, whereas tensile-dominated ones are more suited for applications demanding superior stiffness and load capacity [23].

Widely studied lattice systems include cubic frameworks such as body-centered cubic (BCC) and face-centered cubic (FCC) designs, where nodes are positioned at the center of structures or cube faces, respectively. Variations like (BCCZ) and (FCCZ) integrate vertical elements, offering advantages in terms of both simplicity and material efficiency during production [24]. The hexagonal structure (Hexagonal Close-Packed, HCP) is characterized by nodes arranged in a hexagonal pattern within a plane, with this arrangement repeating across multiple layers. The simple cubic lattice (Simple Cubic, SC) comprises rods or elements connecting nodes located at the vertices of a cube, with each node linked to its neighbouring nodes. This type of structure is primarily utilized as a baseline model or in applications that require a lightweight configuration with minimal load-bearing capacity.

In the study, conducted by Gu et al., the SAMP-1 material model was employed with the calculated damage parameters to simulate the loading–unloading uniaxial tension tests of a talc-filled, impact-modified polypropylene material. The damage parameters were calculated using two documented methods: one based on the loading and unloading moduli, and the other on the volume strain. The two-dimensional DIC optical strain measuring method was applied for strain measurement [7].

Based on the analysis of Bogusz et al. [25] and other literature, two topologies of lattice structure were selected in the current study: the traditional honeycomb (hexagonal) and the auxetic topology of the re-entrant honeycomb. These structures, manufactured using 3D printing with Durable Resin V2, were examined using the 3D DIC method, which is widely used in strength testing, including for numerical simulations [26,27,28,29].

The article [20] examines the mechanical behavior and energy absorption capabilities of three lattice structures, using Finite Element Analysis and DIC to assess strain distribution, which showed a reliable correlation with experimental data. The study [30] applied DIC to analyze strain evolution, crack formation, and failure modes in 3D-printed lattice-reinforced cementitious composites under bending loads.

It should be noted that the typical size of lattice structures poses challenges, or even makes it impossible, to use DIC, as their struts are too thin to effectively measure strain fields. Therefore, the cells of the selected structures were scaled up for DIC testing while striving to maintain their interaction characteristics.

Subsequently, based on the results of the strength tests of the resin, numerical FEM simulations were performed using the SAMP-1 material model in LS-DYNA. From a practical point of view, the commonly used model is Mat 24. However, since thermoplastics are not incompressible during plastic flow, material laws based on von Mises plasticity are not suitable. Therefore, another material model was utilized, the Semi-Analytical Model for Polymers (SAMP-1 with GISSMO failure—Generalized Incremental Stress-State Dependent Damage Model), which was developed specifically for plastics. The SAMP-1 model is an advanced tool for numerical modeling of polymeric materials, including resins. It has been developed to describe the nonlinear properties of materials, accounting for different stress behaviors, such as variations in compression and tension responses. This model captures characteristics such as strain-rate dependency, viscoelastic and viscoplastic behavior, non-isochoric plastic deformation, and pressure dependency of failure strain, as well as the loss of material stiffness due to damage. The model also incorporates material degradation during unloading. The model is also used in structural and crashworthiness simulations of thermoplastics [31,32].

Using SAMP-1 requires comprehensive testing to accurately define material parameters. For instance, in [32] standard tension, compression and shear tests were conducted to create the input data for the SAMP-1 model. In the current study, the SAMP-1 model was calibrated based on DIC measurements obtained from experimental tension and compression tests. A very good agreement was found between the experimental strength tests and their numerical counterparts. The model was then adapted for simulating lattice structures. The study evaluated the energy absorption efficiency, strain distribution, and mechanical behavior of the structures under compression load.

The presented experimental–numerical approach utilizes the DIC optical strain measurement tool and the SAMP-1 material model for polymer simulations, along with a novel proposal to scale individual topology cells for DIC measurements. Optical systems are commonly used in conjunction with numerical simulations, but this approach has not been reported in other studies. The advantages of this method are highlighted in comparison to nanoscale techniques and machine learning approaches, which are increasingly used in numerical modeling.

Nanoscale techniques allow the study of micro- and nanoscale deformations, including soft materials [33], which can be critical for understanding local phenomena in lattice nodes and elements. However, results at the nanoscale can be difficult to interpret in the context of the global structural behavior. Machine learning facilitates rapid data analysis and handles nonlinearities [34] often present in lattice materials, but it can struggle to link results to physical phenomena and is sensitive to data quality, which can lead to poor alignment between numerical and experimental results. In both cases, there is a lack of bulk information and experimental data.

The proposed framework, based on both material and structural experimental investigations, enables the validation of numerical models using DIC-based deformation field imaging, demonstrating its versatility across various topologies and loading conditions.

## 2. Materials and Methods

**Figure 1:**
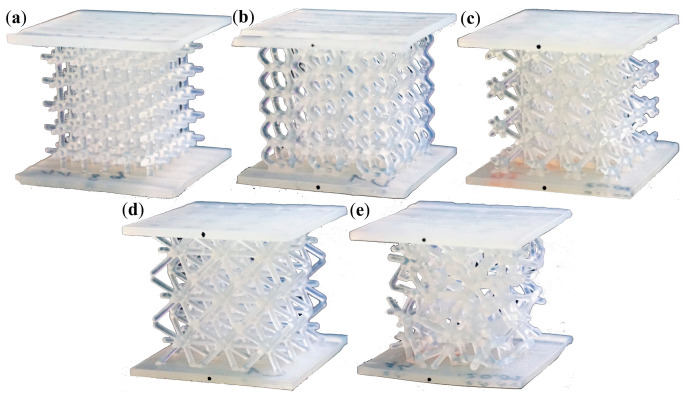
Photographs of the printed lattice structures: (**a**) Grid; (**b**) Hexagon; (**c**) Star; (**d**) Tetra; (**e**) Trabecular [25].

In [25], various configurations of lattice structures were examined. Five types of beam-like structures were analyzed: Grid, Hexagon, Star, Tetra, and Trabecular (Figure 1). The structures were manufactured using stereolithography (SLA). The printing material used was Durable Resin V2, characterized by strong viscoelastic properties. The regular-cell topologies with periodically repeating and interconnected elementary cells were developed. The overall dimensions of the sample were 40 × 40 × 35 mm. The averages of the measured diameters of the beams, of which all structures were composed, were ranging from 1.31 to 1.43 mm, compared to the nominal value of 1.5 mm. The dimension was too small to capture the structural behavior using the available DIC methods.

The evaluated topologies demonstrated unique and versatile characteristics while maintaining a simple design. One example included a topology with quasi-isotropic properties and a quasi-irregular structure, referred to as the Trabecular topology. The rod-shape Grid structure, in which beams were oriented only in two perpendicular directions, exhibited orthotropic characteristics. Other topologies featured beams oriented in various directions and at different angles relative to the applied compression force, as seen in the Star and Tetra topologies. Different stress conditions were observed across the topologies, primarily depending on the number and orientation of the beams. The Hexagonal topology, resembling the honeycomb structure, was highlighted for its efficiency, similar to honeycomb sandwich structures commonly used in the aerospace industry and inspired by nature [25].

**Figure 2:**
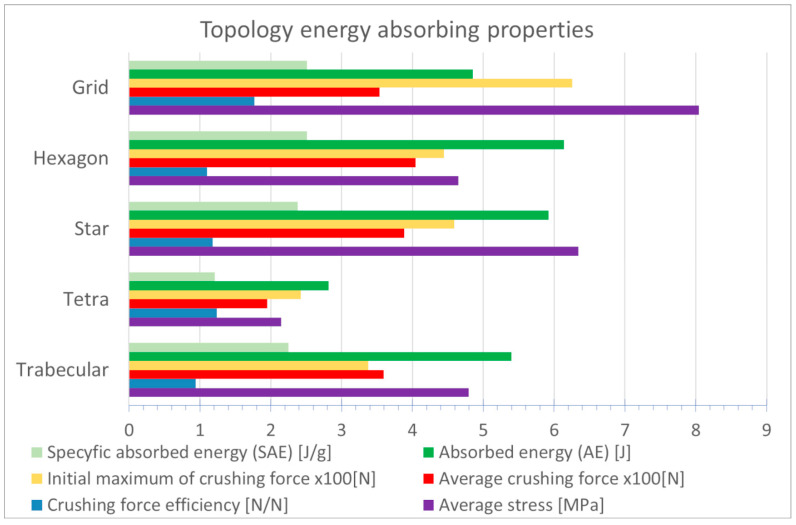
Graphical comparison of the energy-absorbing parameters of the topologies studied [25].

After analyzing the energy-absorbing parameters, it was concluded that the Star and Hexagon topologies achieved the highest absorbed energy (AE), with 6.14 and 5.92 J, respectively—Figure 2. The Trabecular topology also achieved a relatively high AE. The lowest AE of 2.82 J was recorded for Tetra topology. Both the volumes and weights of the topologies tested were similar, which was a condition for allowing a mutual comparison; therefore, the AE and SAE ratios were similar.

The Hexagon topology does not contain beams oriented in the load axis. Therefore, their transition from the linear part is smooth. It presents a considerably smooth course of a force-displacement compression curve. The flat plateau in the central part of the graph is clearly outlined.

The honeycomb structure is an efficient, regular hexagonal lattice known for its exceptional balance between strength, stiffness, and low weight, making it highly versatile across various engineering disciplines. Consequently, it has been included in this study.

**Figure 3:**
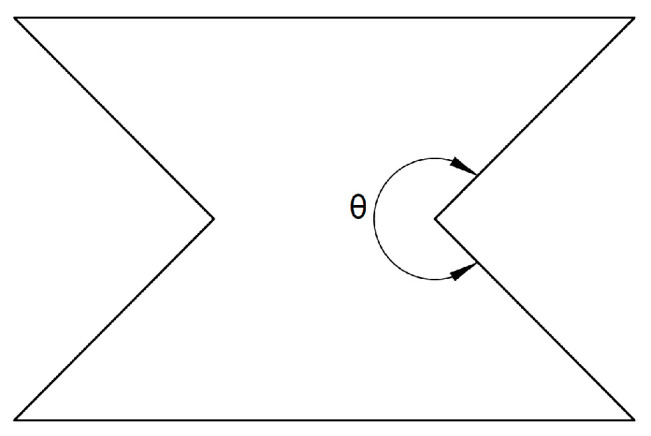
Schematic of the basic cell of the re-entrant honeycomb topology (adapted from [35]).

Another lattice structure selected for investigation is the auxetic re-entrant honeycomb. Auxetic materials and structures are characterized by a negative Poisson’s ratio, which describes the response of a solid to uniaxial stress. It is defined as the negative ratio of transverse strain to axial strain. This dimensionless quantity measures a material’s deformation behavior. One of the earliest models of structures with a negative Poisson’s ratio is the re-entrant honeycomb. The term “re-entrant” refers to shapes containing angles greater than θ = 180° directed inward—Figure 3.

Auxetic structures have a wide range of applications, including biomedical materials, shock absorbers, robotics, sports equipment, energy harvesting devices, filters, textiles, and materials used in the aerospace and construction industries [35].

**Figure 4:**
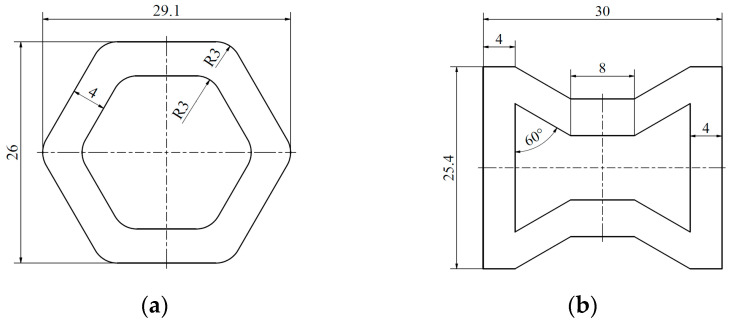
The basic topology cell of the structures: (**a**) honeycomb and (**b**) re-entrant honeycomb.

The energy-absorbing tests on lattice structures presented in this article were performed and analyzed using the Digital Image Correlation (DIC) method. In the first stage of the design, a single enlarged unit cell of each topology was created. The schematics and dimensions of the hexagonal and re-entrant honeycomb cells are presented in Figure 4a and Figure 4b, respectively. The dimensions and proportions of the individual cells in each topology are similar. The thickness of the individual cell struts was set at 4 mm so that when the cells were assembled into a structure, the overall thickness would be 8 mm. The thickness of the cell walls, and thus the entire structure, was also set at 8 mm to prevent buckling.

**Figure 5:**
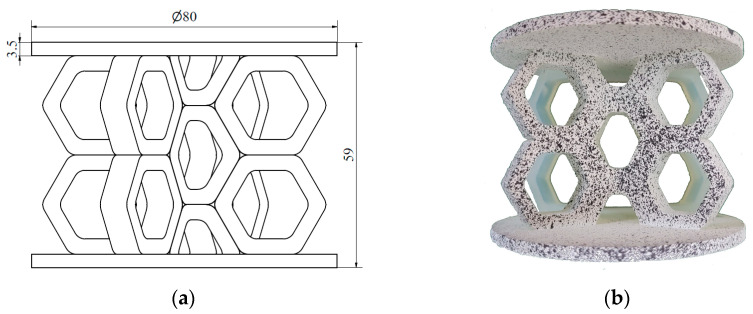
Hexagonal structure loaded along axis 1: (**a**) schematic diagram; (**b**) photograph.

**Figure 6:**
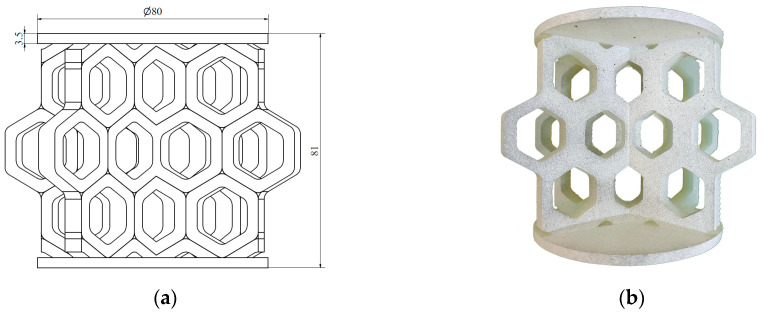
Hexagonal structure loaded along axis 2: (**a**) schematic diagram; (**b**) photograph.

**Figure 7:**
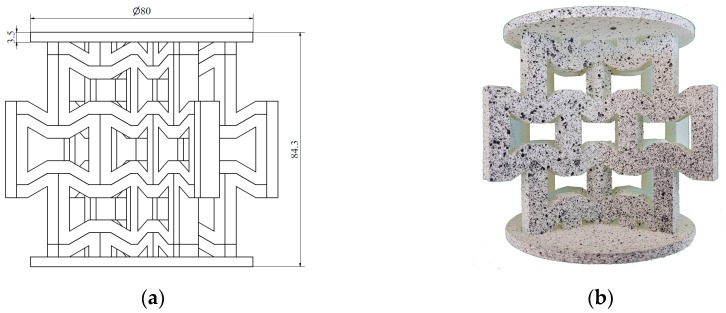
Re-entrant honeycomb structure loaded along axis 1: (**a**) schematic diagram; (**b**) photograph.

**Figure 8:**
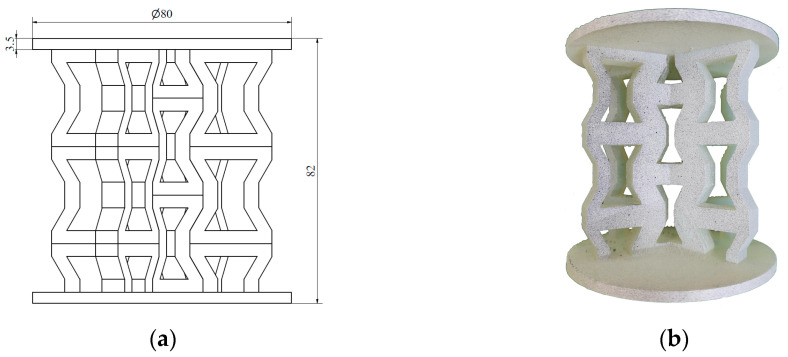
Re-entrant honeycomb structure loaded along axis 2: (**a**) schematic diagram; (**b**) photograph.

In the next step, the individual unit cells were replicated to form a 2D layer of the respective structure, referred to as the wall. Each sample structure consisted of two such walls intersecting at the centre at an angle of 120°. Circular plates with a diameter of 80 mm and a thickness of 3.5 mm were positioned at the top and bottom of the samples to apply the load. Two versions of each type of structure were developed, as the load was applied along two perpendicular axes. The structures with characteristic dimensions (a) and their corresponding photographs (b) are presented in the following figures. Figure 5 and Figure 6 show the hexagonal lattice structure loaded along axes 1 and 2, respectively, while Figure 7 and Figure 8 illustrate the re-entrant honeycomb structure under analogous loading configurations.

The strut thickness and the angle between the walls were designed to facilitate analysis using DIC, minimizing the influence of adjacent layers on the measurements. The angle was selected to ensure that the observed surface was clearly visible to both cameras of the system, allowing the deformation, strain field, and crushing mechanisms to be precisely captured.

The lattice structure specimens required for the study were fabricated using SLA technology on a Formlabs Form 2 printer (Formlabs Inc., Somerville, MA, USA) with light-curable Durable Resin V2 material. This material is characterized by its impact and wear resistance, as well as a low friction coefficient. It is recommended for producing high-impact, rigid yet flexible parts, and for applications requiring low-friction surfaces. It is particularly suitable for prototyping parts that will eventually be made of polypropylene or HDPE (high-density polyethylene). However, its use is not recommended in environments with temperatures exceeding room temperature [36]. The usefulness of this material for prototyping energy-absorbing structures and observing their behavior during failure was confirmed in study [25]. The technical parameters of Durable Resin V2 are provided in Table 1.

In subsequent stages of the printing process, the components were submerged in an isopropyl alcohol bath for 15 min and then subjected to a curing process, which involved annealing combined with UV irradiation. This process was conducted at 60 °C for approximately 60 min, in accordance with the manufacturer’s guidelines. The structures were printed with a full raft and supports in the form of beams, which were manually removed using precision tools.

## 3. Experimental Methods

The prepared specimens of hexagonal honeycomb and auxetic re-entrant honeycomb lattice structures were subjected to axial compression testing. Each type of structure was tested in two loading directions (Figure 5, Figure 6, Figure 7 and Figure 8), with two to three specimens of each type tested.

**Figure 9:**
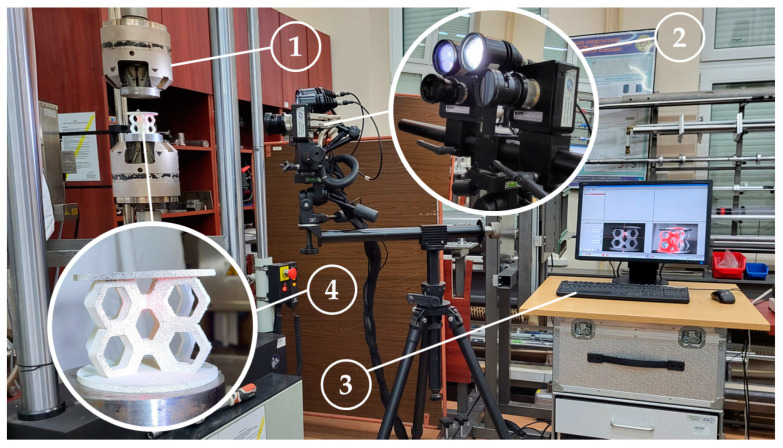
Experimental setup: (1) universal testing machine; (2) two calibrated cameras of the DIC system with lighting; (3) DIC system computer with software; (4) specimen placed on the measurement setup.

The experimental setup shown in Figure 9 consisted of an Instron 8862 testing machine (Illinois Tool Works Inc., Norwood, MA, USA), a pair of GOM Aramis 4M optical system cameras (Carl Zeiss GOM Metrology GmbH, Braunschweig, Germany) with lighting mounted on a tripod, and a computer station equipped with software supporting the Aramis system. Strain maps were measured during loading on the surface of the specimens, which were marked with a stochastic black-and-white pattern, as shown in Figure 9. The system is designed for measuring strain and deformation on a material surface during loading, using two high-resolution CCD cameras (2358 × 1728 pixels) and the digital image correlation method to capture a sequence of three-dimensional images.

The measurement process began with the calibration of the optical system. To ensure full surface coverage of the area of interest, the measurement area was set to 100 mm × 75 mm based on the calibration table. The camera settings were adjusted, including the distance between the sample and cameras (400 mm) and the angle between the lenses (25°). Manual focus lenses with a 50 mm focal length were used. The image capture frequency and signal sampling rate were set to 5 Hz. At the end of the calibration process, reference images were taken to verify calibration accuracy and ensure more than 90% pattern coverage of the area of interest. The distinct areas tracked by the DIC were defined by a facet size of 15 × 15 pixels, corresponding to 0.3 × 0.3 mm. GOM Aramis software v6.2.0-6 (Carl Zeiss GOM Metrology GmbH, Braunschweig, Germany) [37] for calculating strain tensors was used.

The tests were conducted with displacement control for the machine’s crosshead. The loading rate was set to 40 mm/min, and the specimens were compressed to 2/3 of their initial height. If early degradation of the structure occurred, such as cracks or significant buckling, the tests were interrupted. The force data from the testing machine and the strain maps from the optical system were synchronized.

**Figure 10:**
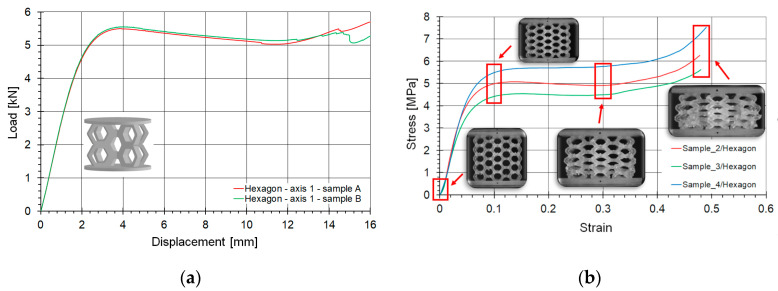
Comprehensive compression curve results for all samples of the hexagonal topology (**a**); compared with the original lattice structure (with non-enlarged cells) based on [25] (**b**).

Comprehensive compression curve results for all samples of the hexagonal lattice topology loaded along axis 1 are shown in Figure 10a. As can be seen, the curves almost overlap, indicating the repeatability of the tests. These curves were visually compared to the results of the original lattice structure (with non-enlarged cells—Figure 10b) tested in [25]. The shape of the curves is preserved, which positively indicates the accuracy of the topology reproduction.

The hexagonal topology is inherently a flat, two-dimensional structure. Enlarging the cells and presenting only two intersecting layers did not significantly affect the nature of the curve. It still exhibits a linear progression in the initial phase, then smoothly transitions into a plateau, and intensifies its growth in the final phase.

To calibrate the LS-DYNA SAMP-1 material model, tensile and compression strength tests were conducted according to standard procedures. These tests were performed using the same optical system employed for analyzing the lattice structures, in conjunction with a ZwickRoell KAPPA 50 DS testing machine (ZwickRoell GmbH & Co. KG, Ulm, Germany). The methodology for the material strength tests closely mirrored that used for the lattice structures.

**Figure 11:**
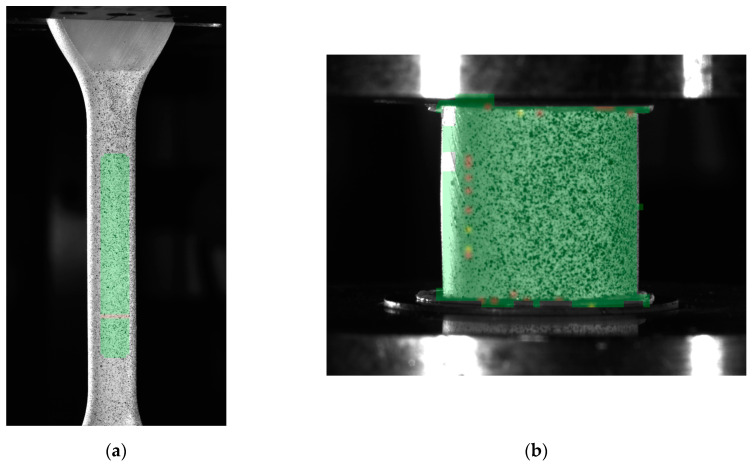
Standard mechanical tests using the DIC system: (**a**) tensile test; (**b**) compression test.

Tensile tests were carried out on specimens with a circular cross-section and a nominal diameter of 6.5 mm (Figure 11a). Longitudinal and transverse strains, as well as the elastic modulus and variation in the Poisson’s ratio function, were measured using the optical system software. These parameters were derived from strain data collected from selected regions of the axial and transverse strain maps, as well as a virtual extensometer developed within the optical system. The actual cross-sectional area of the specimen’s gauge section was also determined, enabling the calculation of true stress based on the following formula:

(1) $$\sigma_{true}=\frac{F_{i}}{A_{i}},$$

where *F_i_* is the current force and *A_i_* is the current cross-sectional area.

True strain was calculated as logarithmic strain. According to the software documentation, deformation *λ* in the GOM Aramis v6.2.0-6 software is defined as [37]:

(2) $$\lambda =\underset{l\to 0}{\lim}\frac{l+∆l}{l},$$

where: *l* is the initial distance and Δ*l* is the increment of distance.

Compression tests were performed on cylindrical specimens with a diameter-to-height ratio of 1:1 (12 × 12 mm, Figure 11b). Strain measurements were recorded using the virtual extensometer. Based on the resulting material curves, parameters essential for the SAMP-1 model were determined.

## 4. Numerical Simulation Procedure

The photopolymer resin used to manufacture the analyzed structures is a plastic material with properties similar to those of polyethylene-based materials. These materials are widely used in a broad range of applications, from packaging to structural components. They are characterized by low density, relatively high tensile strength (22–34 MPa), and significant elongation at break (3–700%). These values may vary depending on processing conditions, additives, and the molecular orientation of the material [38]. The specific characteristic parameters of the Durable Resin V2 were specified in Table 1, in Section 2.

Due to their specific properties, plastics are challenging materials in terms of mathematical description. Accounting for most of the characteristics that define plastics requires advanced constitutive material models. Several models exist that can represent the mathematical behavior of these materials [31,39]. One of the constitutive models frequently used in scientific research is the SAMP-1 model [7,40,41,42].

A semi-analytical model for polymers incorporating necking phenomena, strain rate dependency, unloading behavior damage and failure under complex loading conditions was used. The yield surface is determined by the input data and resembles the von Mises yield surface, when only a tensile curve is provided, but it takes the form of a Drucker–Prager cone if additional data, such as compressive, shear, or biaxial curves, are supplied alongside the tensile curve [43].

The yield surface associated with this constitutive model is mathematically described by the following equation:

(3) $$J_{2}\;-\;A_{0}\;-\;A_{1}I_{1}\;-\;A_{2}I_{1}^{2}=0,$$

where:

*J*_2_—second invariant of deviatoric stress tensor,

*I*_2_—first invariant of the stress tensor,

*A*_0_, *A*_1_, *A*_2_—coefficients determined based on simple experimental tests: tension, compression, and shear [40].

In the presented study, experimental data from tensile and compression tests were used, which limited the yield surface to the Drucker–Prager surface. The missing data for the shear test, required to construct the yield surface, were determined using internal algorithms based on the relationship shown below [39]:

(4) $$\sigma_{s}=\frac{2\sigma_{c}\sigma_{t}}{\sqrt{3}\left(\sigma_{t}+\sigma_{c}\right)},$$

where:

*σ_s_*—yield stress in shear,

*σ_c_*—uniaxial yield stress in compression,

*σ_t_*—uniaxial yield stress in tension.

Material model SAMP-1 provides the capability to define two different flow rules, named associated and non-associated, depending on the definition of the Poisson’s ratio (*ν_p_*) used. If the Poisson’s ratio is provided as a constant input, it corresponds to a non-associated flow rule. In the analyses and considerations presented in this study, an associated flow rule was used, which can be expressed as [40]:

(5) $$g=\sqrt{3J_{2}+\alpha I_{1}^{2}},$$

where *α* is a function of the plastic Poisson’s ratio *ν_p_*:

(6) $$\alpha =\frac{1-2\nu_{p}}{2\left(1+\nu_{p}\right)}.$$

**Figure 12:**
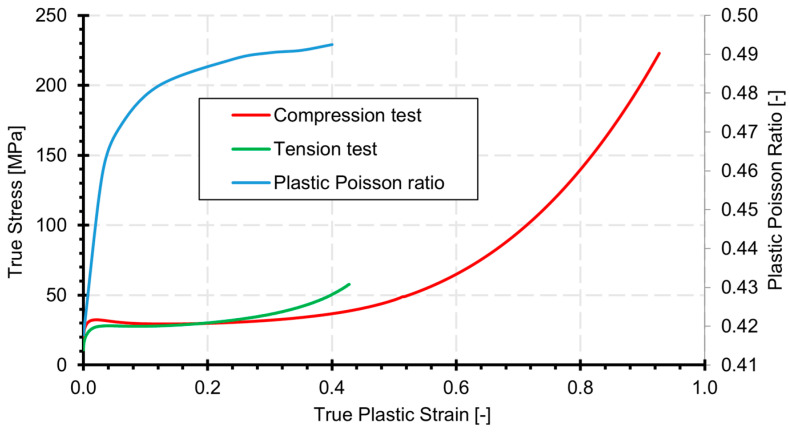
Tabulated input data for the Durable resin V2 material applied to the SAMP-1 constitutive model.

Table 1 in the previous section presents the basic material constants used in the SAMP-1 constitutive model. The remaining material model data were determined based on the experimental tests described in Section 3. The tabulated data are presented in Figure 12.

In the analyses, material curves based on the true stress–true plastic strain relationship, calculated using Formulas (1) and (2), were utilized.

**Figure 13:**
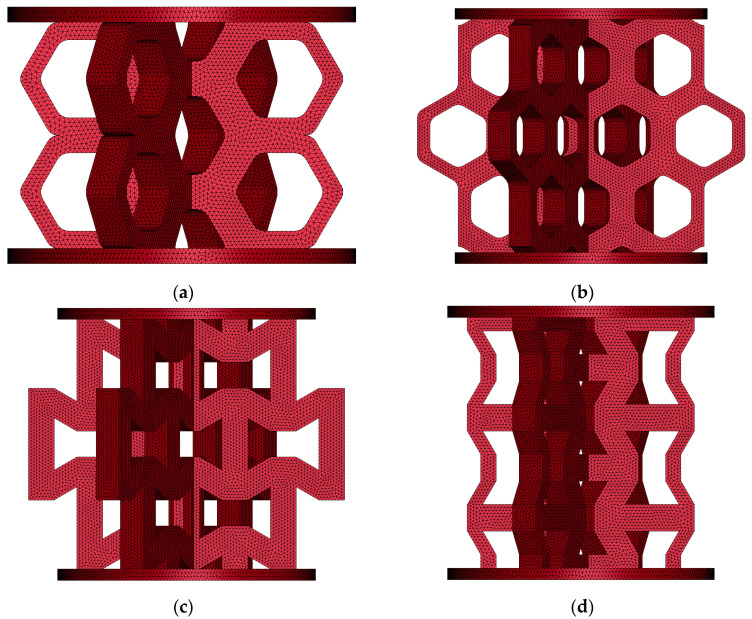
Numerical models of lattice structures: (**a**) hexagonal topology loaded along axis 1; (**b**) hexagonal topology loaded along axis 2; (**c**) re-entrant honeycomb loaded along axis 1; (**d**) re-entrant honeycomb loaded along axis 2.

All numerical analyses, including basic tests and compression tests of lattice structures, were conducted using the LS-DYNA software (version R15.0.2). Dynamic equilibrium equations in FEM form were integrated with the central difference scheme, which is classified as an explicit approach. Although the assumed solution method incorporates dynamic terms, the selected loading rate application made the inertial effect negligible. Constant stress tetrahedron finite elements with one-point Gauss were used in modeling analyzed problems. The geometries of the structures subjected to discretization are presented in Figure 13, [44].

The loading of samples in the basic numerical tests was performed by applying a prescribed displacement curve. For the tensile test sample, displacements were applied to the nodes in the gripping section of the sample, corresponding to the portion held by the jaws of the testing machine. For the compression test sample, its numerical model was positioned between two rigid surfaces. Loading was applied by displacing one of these rigid surfaces. To facilitate the compression simulation, a penalty-based contact interaction was defined between the sample surface and the rigid surface, utilizing the Coulomb friction model with a friction coefficient of *μ* = 0.2.

The basic parameters of the analyses for lattice structures were consistent with the parameters and procedures used for the basic analyses. Additionally, the numerical analyses of lattice structures incorporated material erosion using the GISSMO model with limited damage criteria [39]. Only the failure-type procedure based on the curve of equivalent plastic strain at failure (*ε_pf_*) as a function of stress triaxiality (σ*) was utilized [44].

## 5. Results and Comparisons

### 5.1. Development of the SAMP-1 Numerical Material Model from Experimental Data

Based on the experimental studies conducted, the SAMP-1 material model was developed and calibrated. The calibration process utilized material curves derived from tensile tests (Figure 11a), compression tests (Figure 11b), and the variation in the Poisson’s ratio observed during tensile testing. The experimentally determined curves were compared with those obtained from the numerical model.

**Figure 14:**
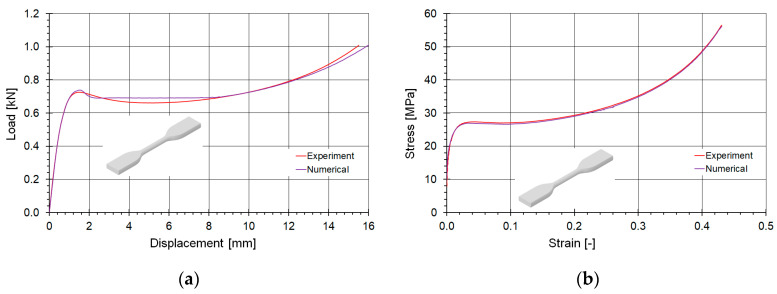
Comparison of the fitting of the tensile material curves determined numerically and experimentally: (**a**) in force-displacement coordinates, and (**b**) true stress–true plastic strain domain.

The comparison of tensile curves is presented in Figure 14 in terms of force-displacement coordinates (a) and true stress–true plastic strain coordinates (b). It was concluded that a very good agreement between the curves was achieved in both reference domains. The linear part of the force curve was accurately captured and within the plateau region the loading level is identical. The average plateau force value differed by only a less than one percent. The overall behavior of the stress–strain curves in the plastic deformation was also identical.

**Figure 15:**
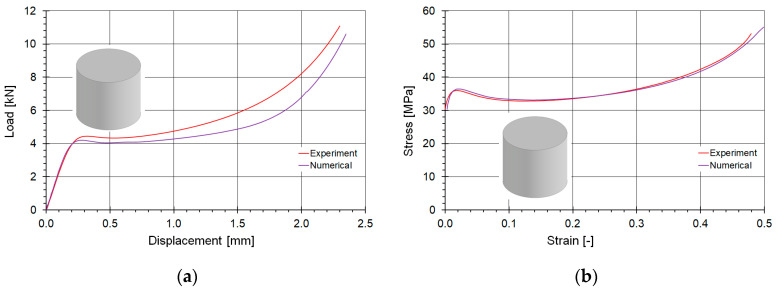
Comparison of the fitting of the compression material curves determined numerically and experimentally: (**a**) in force-displacement coordinates, and (**b**) true stress–true plastic strain domain.

Analogously, Figure 15 shows a comparison of the material compression curves for cylindrical samples. The loading curve fitting can be assessed as good and satisfactory. In this case, the plateau forces are higher in the experiment, whereas, similarly to the tensile tests, the stiffness of the numerical model is identical. The average plateau force value differs by a few percent.

During the calibration process, a reasonable agreement between the model and the experimental results was observed. The characteristics of the experimental curves were accurately replicated in the numerical simulation and subsequently used for evaluating the compression tests of the lattice structures.

### 5.2. Comparison of Compression Curves for the Studied Lattice Structures

**Figure 16:**
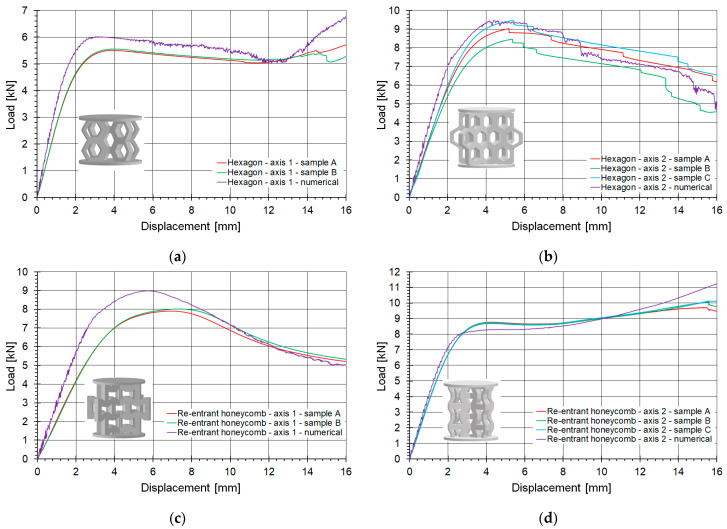
Comparison of compression curves for evaluated topologies, determined numerically and experimentally: (**a**) hexagonal topology loaded along axis 1; (**b**) hexagonal topology loaded along axis 2; (**c**) re-entrant honeycomb loaded along axis 1; (**d**) re-entrant honeycomb loaded along axis 2.

The primary criterion for assessing the accuracy of a numerical model is its agreement with experimental results, and validation is the key method for evaluating the model’s applicability. In this study, experimental and numerical results were compared in various ways. This section focuses on the comparison of compression curves obtained from numerical calculations using the SAMP-1 material model against experimental measurements. The curves were analyzed in the force-displacement domain, as shown in Figure 16.

Figure 16a,b illustrate the behavior of the honeycomb topology subjected to compression along axes 1 and 2, respectively, while Figure 16c,d depict the behavior of the re-entrant honeycomb topology under analogous loading conditions. The presented comparative curves are limited to a shortening of 16 mm. The loading of the structures along the 16 mm displacement path resulted from the initial geometric assumptions, which predefined the effective operation of the structures. Beyond 16 mm, the highly deformed structures became so compressed that strongly contact-dominated phenomena emerged as the primary numerical issue, which was not the main focus of this study.

Table 2 provides a quantitative summary comparing the FEM analyses with the experimental results. It includes the maximum and average force values for all examined topologies and loading conditions. The maximum force is defined as the initial peak observed after the linear part of the curve. In some cases, after a slight drop in the curve within the plateau region, the force may increase even beyond this initial extremum. Additionally, the table presents the average masses of the topology samples and the heights of the structures, excluding the thicknesses of the top and bottom plates.

The hexagonal honeycomb structure subjected to numerical analysis under axis-1 loading demonstrated good agreement with the experimental results. However, it exhibited slightly higher stiffness, with maximum and average force values approximately 8–9% greater than those observed experimentally (Figure 16a). The numerical curve rises significantly more than the experimental curves at the end.

The best agreement with experimental results was achieved for the same honeycomb topology under axis-2 loading (Figure 16b). The force discrepancies were approximately 5%, and the numerical curve closely matched the experimental curve obtained for one of the strongest samples. The slope of the initial linear part of the curve also fell within the range of the experimental curves.

For the re-entrant honeycomb topology tested under axis-2 loading (Figure 16d), very good agreement was observed. According to Table 2, the maximum force differed by approximately 6%. However, throughout most of the curve length, the numerical and experimental curves overlapped, with the difference in average force being below 1.5%.

The weakest agreement was observed for the re-entrant honeycomb topology under axis-1 loading (Figure 16c). After yielding, the maximum force was nearly 13% higher in the numerical model compared to the experiment. However, it is worth noting that the curves converged towards the end of the compression process.

The numerical model demonstrated at least adequate performance in all comparisons. Although the fit is not perfect, the shape of the curves was preserved in the numerical models. According to Table 2, the maximum differences in average force values did not exceed 10 percent. It is noteworthy that various topologies subjected to different loading conditions were considered, highlighting the versatility of the developed model.

### 5.3. Comparison of Failure Mechanisms

**Figure 17:**
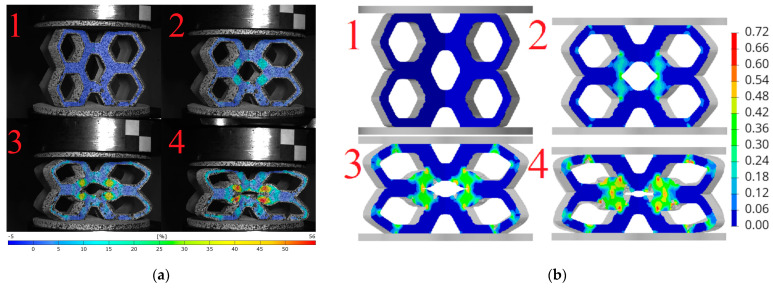
Deformation and failure mechanisms for hexagonal topology loaded along axis 1: (**a**) determined experimentally and (**b**) determined numerically. Numbers 1–4 indicate successive compression stages of the structure.

**Figure 18:**
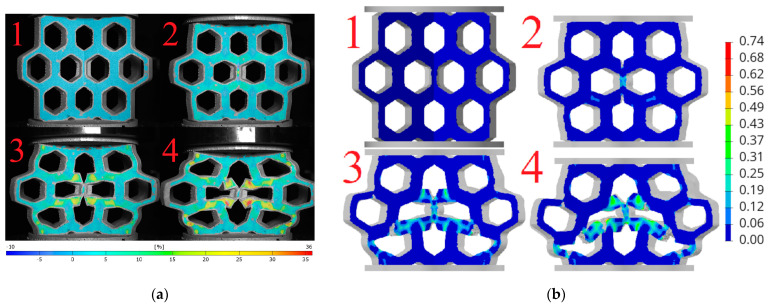
Deformation and failure mechanisms for hexagonal topology loaded along axis 2: (**a**) determined experimentally and (**b**) determined numerically. Numbers 1–4 indicate successive compression stages of the structure. Numbers 1–4 indicate successive compression stages of the structure.

**Figure 19:**
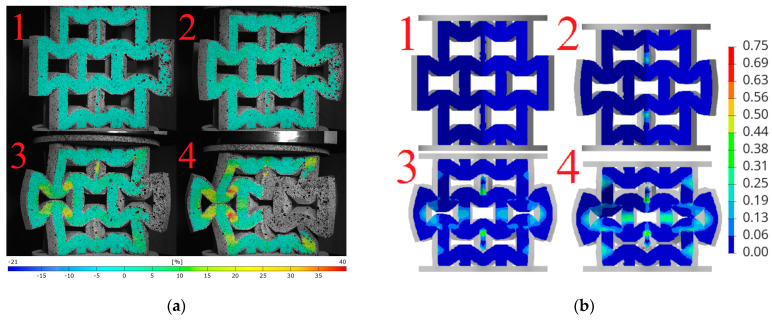
Deformation and failure mechanisms for re-entrant honeycomb topology loaded along axis 1: (**a**) determined experimentally and (**b**) determined numerically. Numbers 1–4 indicate successive compression stages of the structure.

**Figure 20:**
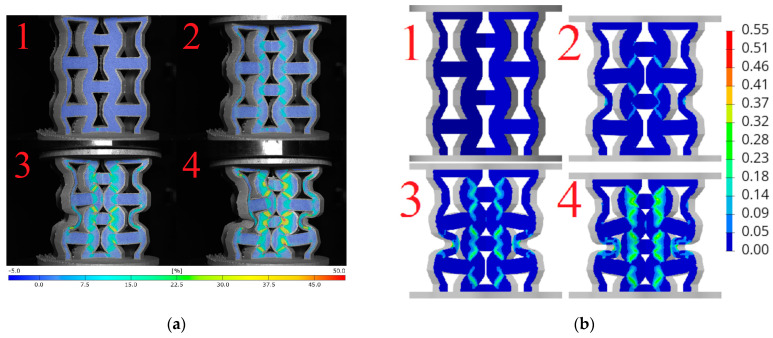
Deformation and failure mechanisms for re-entrant honeycomb topology loaded along axis 2: (**a**) determined experimentally and (**b**) determined numerically. Numbers 1–4 indicate successive compression stages of the structure.

Based on the obtained DIC results, deformation and failure mechanisms were evaluated and compared with numerical simulations for investigated topologies. The hexagonal topologies, studied along both axes, are shown in Figure 17 and Figure 18, while the re-entrant honeycomb topologies for both loading configurations are presented in Figure 19 and Figure 20. Numbers 1–4 indicate successive compression stages of the structures, from the beginning to the 16mm compression phase. All images for each experimental data were generated using the DIC system. Engineering major strain maps are presented in the left column, with the corresponding numerical results displayed in the right column. These maps were presented at equal time intervals, every 16 s, which corresponds to the sample shortening of 5.3 mm. Uniform strain scales were applied for both the DIC tests and the FEM simulations.

The analysis of the hexagonal topology loaded along axis 1, presented in Figure 17a, indicates that the highest strain values occur in the central part of the structure, specifically on the diagonal struts of the central honeycomb cell. These regions, four relatively symmetrically distributed areas, are typical of plastic deformation and were interpreted as indicative of bending behavior. The structure experienced plastic deformation, with the honeycomb cells flattening along the loading axis while expanding in width. In the final phase of compression, fractures were observed in the horizontal struts adjacent to the supports.

The simulation shown in Figure 17b reveals that the concentration of strain and the occurrence of fractures are located in the same regions as in the experiment. The highest strain concentrations occurred on the diagonal struts, corresponding to the four symmetrically distributed regions visible in the DIC measurements.

The analysis of the hexagonal topology loaded along axis 2 (Figure 18a) indicates that the highest strain regions were concentrated in the central part of the structure, specifically on the diagonal struts. These correspond to four symmetrically distributed regions of plastic deformation. The major strain values exceeded 35%. After 7 mm of compression, all samples began to fracture in the central region, with the vertical strut delaminating into two halves.

In the numerical analysis, the sample deformed in a similar manner, reaching comparable strain levels in the characteristic regions. The model did not buckle during compression and behaved similarly to the 3D-printed structure.

Analyzing Figure 19a with the re-entrant honeycomb topology loaded in the 1 axis, it can be observed that the sample buckled asymmetrically. As a result of this buckling, the outer regions of the measurement areas were significantly displaced, moving beyond the calibrated working space of the DIC system. This displacement caused a loss of focus, preventing calculations in these areas. The strain map analysis indicates that the highest strain levels occurred on the diagonal inner struts of the outer cells, resulting in plastic deformation of the structure. The strain distribution in regions of maximum values was interpreted as primarily resulting from shear.

In the simulation, presented in Figure 19b, the structure buckled symmetrically. The strain concentrations on the simulated strain maps appeared in different locations compared to the experimental results. In the numerical model, the vertically loaded struts were found to be more stressed, whereas in the experiment, these regions experienced less stress.

The highest stress concentrations for the re-entrant honeycomb topology loaded along the 2-axis occurred on the internal diagonal struts (Figure 20a). In the initial phase of the experiment, the expected auxetic behavior of the sample was observed. It is evident from the presented pictures that as vertical compression increases, the sample also narrows along the horizontal axis.

Figure 20b presents an analogous series of four images of numerical calculation. The strain concentrations in the simulation were located in the same regions as in the experiment, specifically on the internal diagonal struts of the cells. During compression, the model did not buckle and behaved similarly to the 3D-printed structure.

It was noted that during the compression of all the above structures, a complex stress state occurs. In the hexagonal topology, bending plays a significant role in the regions with the largest major strains. Plastic hinges form in these areas, where the strains are large and approach crushing levels. This ultimately leads to the plastic deformation of the structure. In the re-entrant honeycomb structure, shear deformations dominate in the most heavily loaded regions.

**Figure 21:**
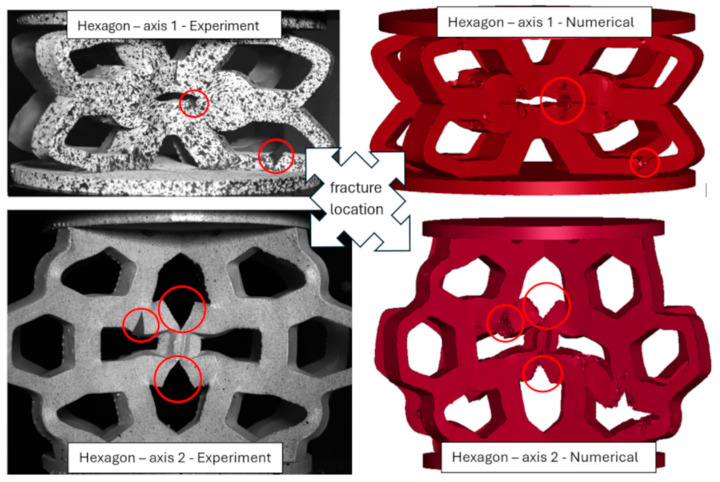
Comparison of the fracture locations for the hexagonal structure loaded along axis 1 (top row) and axis 2 (bottom row): experimental studies (left column) and numerical calculations (right column). The areas of structure failure are highlighted with red circles.

During the loading of the hexagonal topology along both perpendicular axes (Figure 21), fractures were observed. The locations of these fractures were compared with corresponding numerical models at a displacement of 16 mm. In both experimental and numerical studies, the fracture locations were marked with circles in Figure 21. The observed fracture patterns were highly similar in both approaches. These fractures occurred at geometric notches, where strain accumulates during loading. The discontinuities formed under conditions of tensile and bending stresses within the structural elements. The numerical failure model, which removes finite elements upon reaching critical strain values, was configured to initiate failure only under positive triaxiality conditions, excluding element destruction caused by complex compressive loads.

For the hexagonal structure loaded along axis 1, experimental studies showed that the first fracture occurred in the lower part of the printed model at a global displacement of 8 mm. The second fracture, located in the central part of the structure, occurred at a displacement of 13 mm. Due to the static loading rate and the direction of the applied load, the force-displacement graph (Figure 16a) does not show fracture initiation as a force drop. In the numerical analyses, fracture initiation occurred almost simultaneously, at 6% effective plastic strain and a von Mises stress of 24 MPa. Although the numerical model does not perfectly replicate the sequential order of fracture initiation, this discrepancy does not significantly impact the overall failure behavior of the structure.

For the hexagonal structure loaded along axis 2, the first fractures appeared as two vertical cracks in the central part of the sample, occurring at a global displacement of approximately 5 mm. The second visible fracture occurred between 11 and 14 mm of displacement (observed across three samples). In this case, the force-displacement graph (Figure 16b) clearly shows fracture initiation as a force drop. Numerically, the first fracture occurred at approximately 4% effective plastic strain and a von Mises stress of 24 MPa, while the second fracture occurred at 16% effective plastic strain and a von Mises stress of 25 MPa. The sequence of fracture formation in the numerical simulations aligned well with the experimental results. The failure characteristics and the force-displacement response of the numerical model closely matched the experimental observations, validating the constitutive model.

For the re-entrant honeycomb structures loaded along both axes, no failure was observed, which is consistent with the results of the experimental tests.

### 5.4. Comparison of Major Strain Curves

DIC measurements allow for the determination of the full displacement and strain field visible to the stereoscopic system. By knowing the displacement field, any type of strain can be calculated: engineering, logarithmic (treated here as true), or Green’s strains [37]. Strain can also be calculated for any measurement area. This advantage of optical displacement measurement tools was utilized in the present study to compare quantitatively the obtained results.

**Figure 22:**
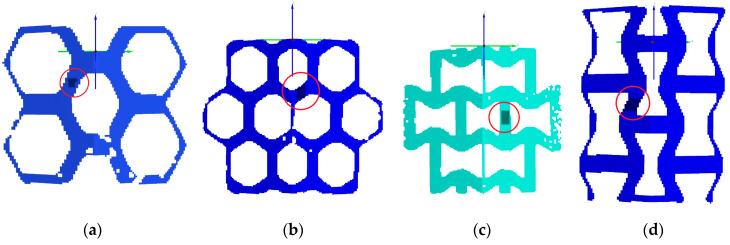
Selection of areas with the highest major strain marked within red circles: (**a**) hexagonal topology loaded along axis 1; (**b**) hexagonal topology loaded along axis 2; (**c**) re-entrant honeycomb loaded along axis 1; (**d**) re-entrant honeycomb loaded along axis 2.

In order to evaluate the fitting of numerical models to experimental results, maps of the engineering major strains were generated in the selected areas of the investigated lattice structures (marked with dark rectangles within red circles in Figure 22). For these areas, graphs were created to show the changes in the major strains as a function of displacement (the sample shortening). The areas were chosen in regions with the most stressed areas. The strain in each point was determined for a rectangular area of approximately 6x6 facets and then averaged.

**Figure 23:**
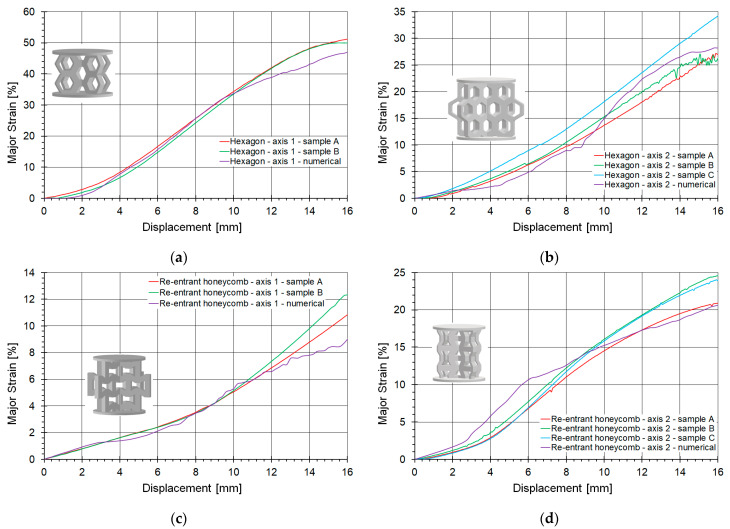
Comparison of the fitting of the logarithmic major strain versus displacement for evaluated topologies, determined numerically and experimentally: (**a**) hexagonal topology loaded along axis 1; (**b**) hexagonal topology loaded along axis 2; (**c**) re-entrant honeycomb loaded along axis 1; (**d**) re-entrant honeycomb loaded along axis 2.

The comparative results for the studied topologies under both loading conditions are presented in four graphs in Figure 23. Each graph includes a numerical curve engineering major strain alongside curves obtained from experimental tests conducted on different samples to illustrate the variation in results. The experimental curves are displayed in red, green, and blue, while the numerical curve is represented in purple. Table 3 summarizes the maximum and average major strain values derived from the presented curves, facilitating a quantitative comparison between the accuracy of the FEM model and the DIC measurements.

The presented numerical curves of major strain behavior demonstrated very good agreement with experimental results for the hexagonal structure under loading along axis 1 (Figure 23a). The difference between DIC and FEM measurements was approximately 7%. The maximum strain at a shortening of 16 mm reached about 50% (Table 3). For the same topology loaded along the perpendicular axis (axis 2), the strain at the same shortening was below 30%. It is worth noting that the first topology was significantly shorter than the others, and a 16 mm shortening constituted about 30% of its height, compared to approximately 20% for the other configurations. The strain curve falls within the range of experimental data but exhibits slight undulations (Figure 23b). The lowest errors were recorded for the hexagonal topology, especially in the case of loading in axis 2 (Figure 23d).

The re-entrant honeycomb topologies also performed well (Figure 23c). The numerical model accurately captured the behavior of this structure when compressed along axis 1. Compression along axis 2 in the numerical simulation produced a slightly undulating curve. The initial segments of the curves were well simulated for all topologies. The differences in average major strains did not exceed 10% (Table 3).

## 6. Summary and Conclusions

This study presents extensive experimental and numerical investigations of selected lattice structure topologies. Two energy-efficient structures, specifically the hexagonal honeycomb and re-entrant honeycomb, were studied. The study also includes the methodology for using the DIC optical system to measure strains and deformations. The aim was to apply a modern FEM numerical model, developed for the analysis of plastics, to simulate energy-absorbing structures made from light-curable Durable Resin V2. The numerical simulations employed the SAMP-1 model, which was calibrated based on experimental tensile and compression tests, from which material curves and variations in Poisson’s ratio were determined.

The experimental compression curve measurements were enhanced using a 3D optical strain measurement technique. The lattice cells of the structures were scaled and optimized for DIC measurements, providing detailed visualizations of structural behavior, failure mechanisms, and strain distributions. The structures were tested in two perpendicular loading directions. The numerical models provided analogous results, allowing for a detailed comparison of both quantitative and qualitative aspects.

Based on the conducted investigations, the following conclusions were drawn:

- During the calibration process, the characteristics of the experimentally determined tensile and compression curves were successfully reproduced in the numerical simulations. The numerical material model SAMP-1 accurately captured the behavior of Durable Resin. A reasonable agreement was observed between the model’s behavior and the experimental strength test results.
- Deformation and failure mechanisms of the lattice structures were evaluated. Various topologies subjected to different loading conditions were considered, highlighting the versatility of the developed model The use of enlarged cells in the studied topologies enabled a detailed DIC analysis of the structural behavior during the compression process. A good agreement was observed between the numerical modeling results and the experimental behavior. The level of agreement varied among different topologies, with the best match achieved for the hexagonal structure compressed along axis 2, where the numerical model’s accuracy for the average force error was below 5%.
- It was noted that during the compression of all considered structures, a complex state of loading occurred. In the hexagonal topology, bending plays a significant role in the regions with the largest major strains. Plastic hinges form in these areas, where the strains are very large and approach crushing levels. In the re-entrant honeycomb structure, shear deformations dominate in the most stressed regions. For axis 2, auxetic behavior was observed for this structure, consistent with the actual behavior.
- In order to confirm the consistency of the numerical calculations with the experimental results, a comparison of the major strain in the areas of maximum stress was performed. Good agreement between the numerical calculations and experimental results was also achieved here. However, the agreement varied for different structures. The hexagonal structure, loaded along axis 2, achieved the best fit, with differences remaining within a range of up to 5%. The weakest fit was observed for the re-entrant honeycomb structure, especially loaded in the axis 1.
- A strain-based failure model was employed in the numerical analyses of both topologies, which satisfactorily captured the failure process of the structures and accurately reflected their global behavior. Failure was observed exclusively in the hexagonal structure (both axes), with fractures occurring in regions of strain and stress concentration, as expected. While the model was active for all four structures, no failure was detected in the re-entrant honeycomb structures, consistent with the experimental results.

The planned continuation of the research will focus on further refining the SAMP-1 numerical model to improve its alignment with experimental results. Additionally, the SAMP-1 model will be used to simulate other topologies, such as those with an increased number of corners or rod-shape morphology, including those developed and analyzed in [25] (Figure 1). A particularly interesting and complex challenge is the application of the model to simulate advanced 3D structures. Examples of such advanced lattice architectures include graded lattice structures or triply periodic minimal surface (TPMS) geometries, all of which are known for their unique mechanical properties and energy absorption capabilities. These efforts will contribute to advancing the subject of this study by enhancing the numerical model and exploring new structural topologies.

## Figures and Tables

**Table 1:** Technical parameters for UV-curable Durable Resin V2 [36].

Properties	Literature Values [36]	Experimental Values	Standard Deviation
Tensile strength [MPa]	28	25	0.3
Tensile modulus [MPa]	1000	999	6
Shear modulus [MPa]	-	353	4
Elongation [%]	55	58	2
Heat Deflection Temperature HDT [°C]	41	-	-
Liquid density [g/cm^3^]	1.06	-	-
Cured density [g/cm^3^]	1.13	-	-

**Table 2:** Summary of compression curves analysis for all topologies.

Sample	High [mm]	Mass [g]	Load (max) [kN]	ΔLoad (max) [%]	Load (avg) [kN]	ΔLoad (avg) [%]
Hexagon_axis 1—exp. (avg)	52.0	70.40	5.53	8.88	4.95	9.03
Hexagon_axis 1—num.		-	6.02		5.40	
Hexagon_axis 2—exp. (avg)	74.0	100.67	8.99	5.56	7.01	4.17
Hexagon_axis 2—num.		-	9.49		7.31	
Re-entrant honeycomb_axis 1—exp. (avg)	77.3	114.60	7.96	12.93	6.07	8.98
Re-entrant honeycomb_axis 1—num.		-	8.98		6.61	
Re-entrant honeycomb_axis 2—exp. (avg)	75.0	93.28	8.74	6.93	8.30	1.40
Re-entrant honeycomb_axis 2—num.		-	8.14		8.41	

**Table 3:** Summary of major strain for all topologies.

Sample	Sample Hight Reduction (16mm) [%]	Major Strain (16 mm) [%]	Δ Major Strain (16 mm) [%]	Major Strain (avg) [%]	Δ Major Strain (avg) [%]
Hexagon_axis 1—exp. (avg)	30.8	50.71	7.42	25.15	6.01
Hexagon_axis 1—num.		46.95		23.64	
Hexagon_axis 2—exp. (avg)	21.6	29.53	4.37	12.56	5.17
Hexagon_axis 2—num.		28.24		11.91	
Re-entrant honeycomb_axis 1—exp. (avg)	20.7	11.60	22.36	4.46	9.91
Re-entrant honeycomb_axis 1—num.		9.01		4.02	
Re-entrant honeycomb_axis 2—exp. (avg)	21.3	23.23	11.29	11.27	1.99
Re-entrant honeycomb_axis 2—num.		20.61		11.49	

## Data Availability

The original contributions presented in the study are included in the article; further inquiries can be directed to the corresponding author.
